# Uric Acid Induces Renal Inflammation via Activating Tubular NF-κB Signaling Pathway

**DOI:** 10.1371/journal.pone.0039738

**Published:** 2012-06-25

**Authors:** Yang Zhou, Li Fang, Lei Jiang, Ping Wen, Hongdi Cao, Weichun He, Chunsun Dai, Junwei Yang

**Affiliations:** Center for Kidney Disease, 2nd Affiliated Hospital, Nanjing Medical University, Nanjing, Jiangsu Province, China; Friedrich-Alexander University Erlangen, Germany

## Abstract

Inflammation is a pathologic feature of hyperuricemia in clinical settings. However, the underlying mechanism remains unknown. Here, infiltration of T cells and macrophages were significantly increased in hyperuricemia mice kidneys. This infiltration of inflammatory cells was accompanied by an up-regulation of TNF-α, MCP-1 and RANTES expression. Further, infiltration was largely located in tubular interstitial spaces, suggesting a role for tubular cells in hyperuricemia-induced inflammation. In cultured tubular epithelial cells (NRK-52E), uric acid, probably transported via urate transporter, induced TNF-α, MCP-1 and RANTES mRNA as well as RANTES protein expression. Culture media of NRK-52E cells incubated with uric acid showed a chemo-attractive ability to recruit macrophage. Moreover uric acid activated NF-κB signaling. The uric acid-induced up-regulation of RANTES was blocked by SN 50, a specific NF-κB inhibitor. Activation of NF-κB signaling was also observed in tubule of hyperuricemia mice. These results suggest that uric acid induces renal inflammation via activation of NF-κB signaling.

## Introduction

A growing number of epidemiologic and experimental evidence suggests that uric acid is an independent risk fact for cardiovascular and renal disease [1], [2], [3]. For patients with hypertension, heart failure or diabetes, hyperuricemia always predicts a high mortality [4], [5], [6]. The mechanisms by which uric acid causes organ injury are still incompletely understood. However, increasing evidence suggests that uric acid is an agent of inflammation [1], [7], [8]. It is also well accepted that monosodium urate crystals stimulated gouty inflammation [9], [10]. Moreover, under sterile conditions, several endogenous substances, especially uric acid plays an important role in mediating inflammation stimulated by pathologic insult and tissue damage [11], [12]. It is conceivable that uric acid could elicit inflammation in kidney.

Renal infiltration of T cells and macrophages in interstitial spaces contributes to the initiation and progression of renal diseases [13], [14]. Inflammatory cells release pro-inflammatory chemokines and cytokines, which thereby lead to a self-aggravation of primary insults. In addition, production of pro-fibrotic cytokines may promote tissue fibrosis by activating matrix-producing effecter cells, such as tubular cells and fibroblasts. Our previous investigation showed that uric acid increase matrix protein fibronectin expression in tubular cells of hyperuricemia mice [15]. Not surprisingly, hyperuricemia increase the risk of renal injury via inflammation in clinical settings. NF-κB signaling, as a key transcription factor, is known to mediate inflammation by regulating the expression of cytokines and chemokines [14]. Moreover, studies have shown that uric acid could activate NF-κB signaling in proximal tubular epithelial cells [15], [16]. However, the mechanism by which uric acid induces inflammation in renal tubule remains unknown.

In this study, we demonstrated that infiltration of T cells and macrophages were markedly increased in hyperuricemia mice kidney, accompanied by an up-regulation of TNF-α, MCP-1 and RANTES expression. Infiltrated cells were largely localized to tubular interstitial spaces. In tubular epithelial cells (NRK-52E), uric acid treatment induced TNF-α, MCP-1 and RANTES mRNA as well as RANTES protein expression. Culture media of NRK-52E cells incubated with uric acid showed a chemo-attractive ability to recruit macrophages. Uric acid activates NF-κB signaling in tubular cell. Moreover the up-regulation of RANTES was blocked by specific NF-κB inhibitor. Evidence of a hyperuricemia-induced activation of NF-κB signaling was also obtained in tubule of hyperuricemia mice. These results suggest that uric acid induces renal infiltration of inflammatory cells and tubule expression of inflammatory mediators via activation of NF-κB signaling.

## Results

### Uric acid induces inflammatory cells infiltration in renal tubular interstitial spaces

**Figure 1:**
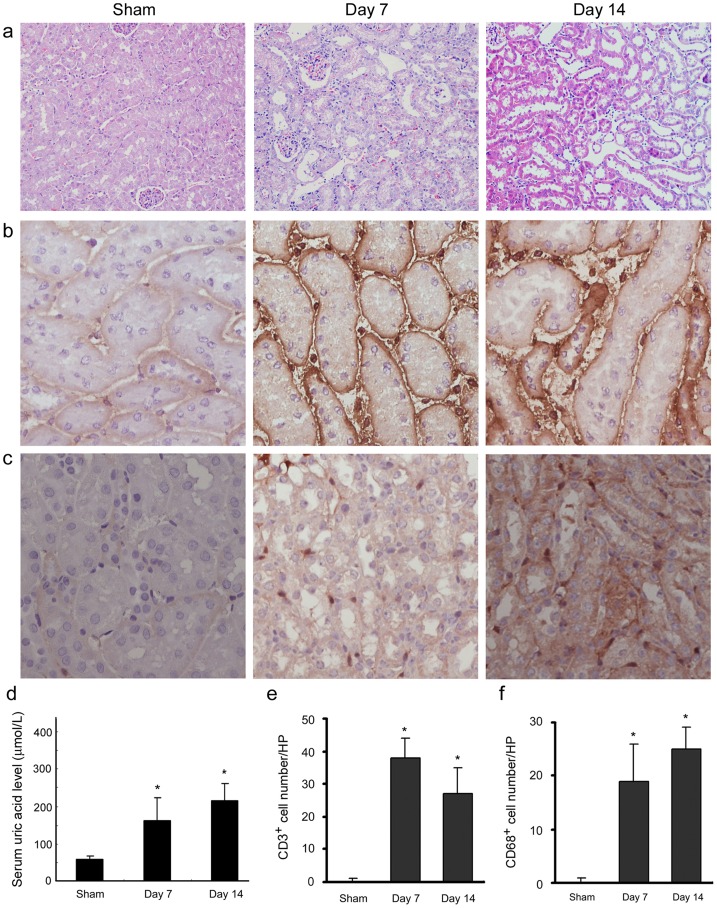
Uric acid induces renal infiltration of T cells and macrophages. (a) Representative micrographs show kidney morphology by H/E staining. (b) Immunohistochemical staining revealed an increased infiltration of CD3+ T cells in kidney of hyperuricemia mice after continuous injection of 7d and 14d, respectively. (c) Immunohistochemical staining revealed an increased infiltration of CD68+ macrophage in kidney of hyperuricemia mice after continuous injection of uric acid for 7d and 14d, respectively. (d) Mice were daily injected with uric acid intraperitoneally. At different time points, as indicated, serum was collected and serum urate levels (μmol/L) were determined. Data are presented as mean ± SEM from five animals per group at each time point, *P<0.05 versus sham (e and f) Graphic presentations of quantitative data. Positive cell numbers per high-power field (×400) were counted. Ten randomly selected field of each kidney were counted. Data are mean ± SEM of five animals per group. *P<0.05 versus sham-control.

Figure 1a shows that the continuous intraperitoneally injection of uric acid induced renal morphological changes, characterized by dilated tubules, expanded interstitial spaces and increased cell number. To assess whether uric acid induced inflammatory cells' infiltration, immunohistochemical staining for CD3 and CD68 were applied. As compared with sham-control group (figure 1b), continuous injection of uric acid caused a markedly increase of T cell infiltration in kidneys at 7d. The renal infiltration of CD3+ T cell is persistent till 14d. Similarly, figure 1c shows that uric acid instigated infiltration of CD68 positive macrophages in renal tubular interstitial spaces. Figure 1d shows the change of serum uric acid level in hyperuricemia mice after intraperitoneally injection of uric acid.

### Uric acid induces renal expression of inflammatory cytokines and chemokines

**Figure 2:**
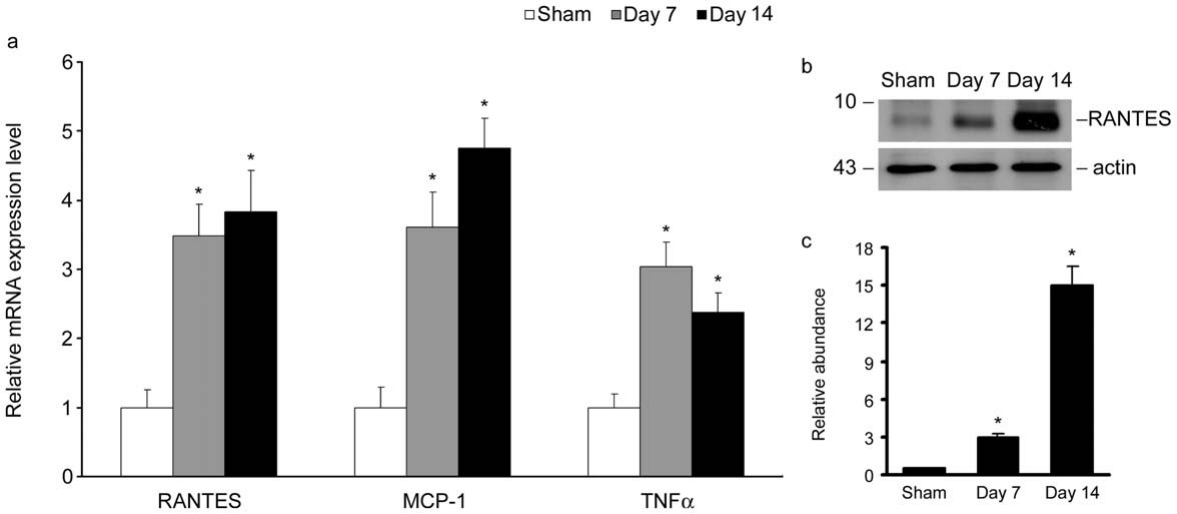
Uric acid induces RANTES, MCP-1 and TNF-α expression in hyperuricemia mice kidneys. (a) Q-PCR results showed that renal RANTES, MCP-1 and TNF-α mRNA expression were increased in kidney of hyperuricemia mice after continuous injection of uric acid for 7d and 14d, respectively. *P<0.05 versus sham-control. (b) Western blot results showed that renal RANTES protein expression was increased in hyperuricemia mice kidneys. (c) Graphic presentation of relative RANTES protein abundance normalized to actin. *P<0.05 versus control (*n* = 5).

We next examined the expression of RANTES and MCP-1, pro-inflammatory chemokines inciting infiltration of T cells and macrophage, in hyperuricemia mice kidneys. As shown in figure 2a, quantitative PCR (Q-PCR) analysis revealed that approximately five and four-fold increase of RANTES and MCP-1 mRNA expressions were observed in mice kidneys after 7days' injection, respectively, when compared with sham-control group. We also examined RANTES protein expression in hyperuricemia mice kidneys by western blot. Figure 2b shows that RANTES protein expression was markedly increased after induction of hyperuricemia, which were consistent with the mRNA results.

We further investigated the expression of TNF-α, an inflammatory cytokine probably produced by infiltrated cells in hyperuricemia mice kidneys. As presented in figure 2a, measured by Q-PCR assay, TNF-α expression was also markedly increased at both 7d and 14d.

### Uric acid induces inflammatory cytokines and chemokines expression in tubular cells

**Figure 3:**
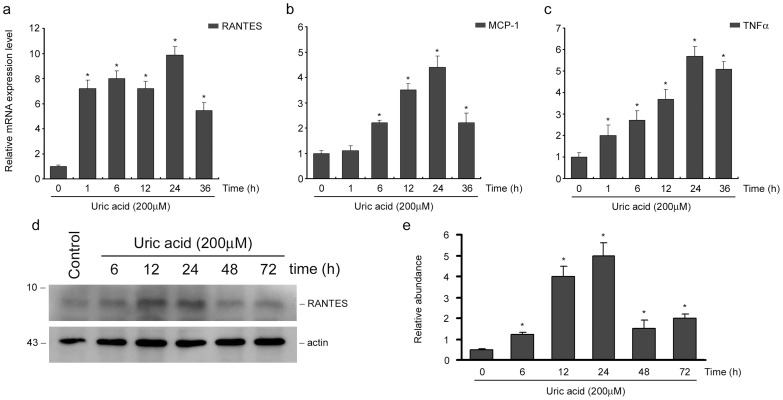
Uric acid induces RANTES, MCP-1 and TNF-α expression in tubular epithelial cells. (a through c) Q-PCR results showed that RANTES, MCP-1 and TNF-α mRNA expression were increased in NRK-52E cells after uric acid treatment for different time periods as indicated. *P<0.05 versus control. (b) Western blot results showed that RANTES protein expression was increased in NRK-52E cells after uric acid treatment. (c) Graphic presentation of relative RANTES protein abundance normalized to actin. *P<0.05 versus control (*n* = 3).

Since the infiltrated cells accumulated in tubular interstitial spaces, we next examined the effects of uric acid on renal tubular epithelial cells in vitro. NRK-52E cells were incubated with uric acid at a concentration of 200μM for various times as indicated. The expression of RANTES, MCP-1 and TNF-α were examined. As shown in figure 3a through 3c, uric acid markedly induced RANTES, MCP-1 and TNF-α mRNA expression. Further, RANTES protein expression was also increased in NRK-52E cells (figure 3d).

### Blockage of urate transporters inhibits the induction of RANTES, MCP-1 and TNF-α

**Figure 4:**
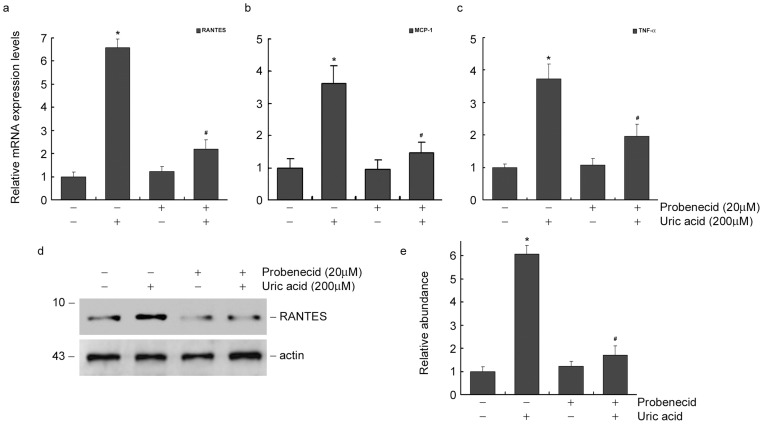
Blockage of urate transporter by probenecid inhibits uric acid-induced RANTES, MCP-1 and TNF-α expression in tubular epithelial cells. (a through c) Q-PCR results showed that probenecid inhibits uric acid-induced RANTES (a), MCP-1 (b) and TNF-α (c) mRNA expression in NRK-52E cells. *P<0.05 versus control. #*P*<0.05 versus uric acid treated cells without probenecid incubation (*n* = 3). (d) Western blot results showed that probenecid inhibits uric acid-induced RANTES protein expression in NRK-52E cells. (e) Graphic presentation of relative RANTES protein abundance normalized to actin. *P<0.05 versus control. #*P*<0.05 versus uric acid treated cells without probenecid incubation (*n* = 3).

Probenecid is a non-specific inhibitor of urate transporters on the membrane of tubular epithelial cells, which has been widely administered in hyperuricemia patients. We therefore reduced the accumulation of uric acid in tubular cells by blocking its transporters and then examined whether the induction of RANTES, MCP-1 and TNF-α was inhibited. As shown in figure 4, Q-PCR analysis demonstrated that probenecid (20μM) inhibited RANTES, MCP-1 and TNF-α mRNA expression induced by 12h of uric acid treatment. RANTES protein expression induced by 24h of uric acid treatment was also abolished. Hence, it suggested that urate transporters blockage reduced the accumulation of uric acid in tubular cells, and therefore abolished the induction of RANTES, MCP-1 and TNF-α.

### Modulation of urate transporter expression regulates tubular express of RANTES, MCP-1 and TNF-α in response to uric acid treatment

**Figure 5:**
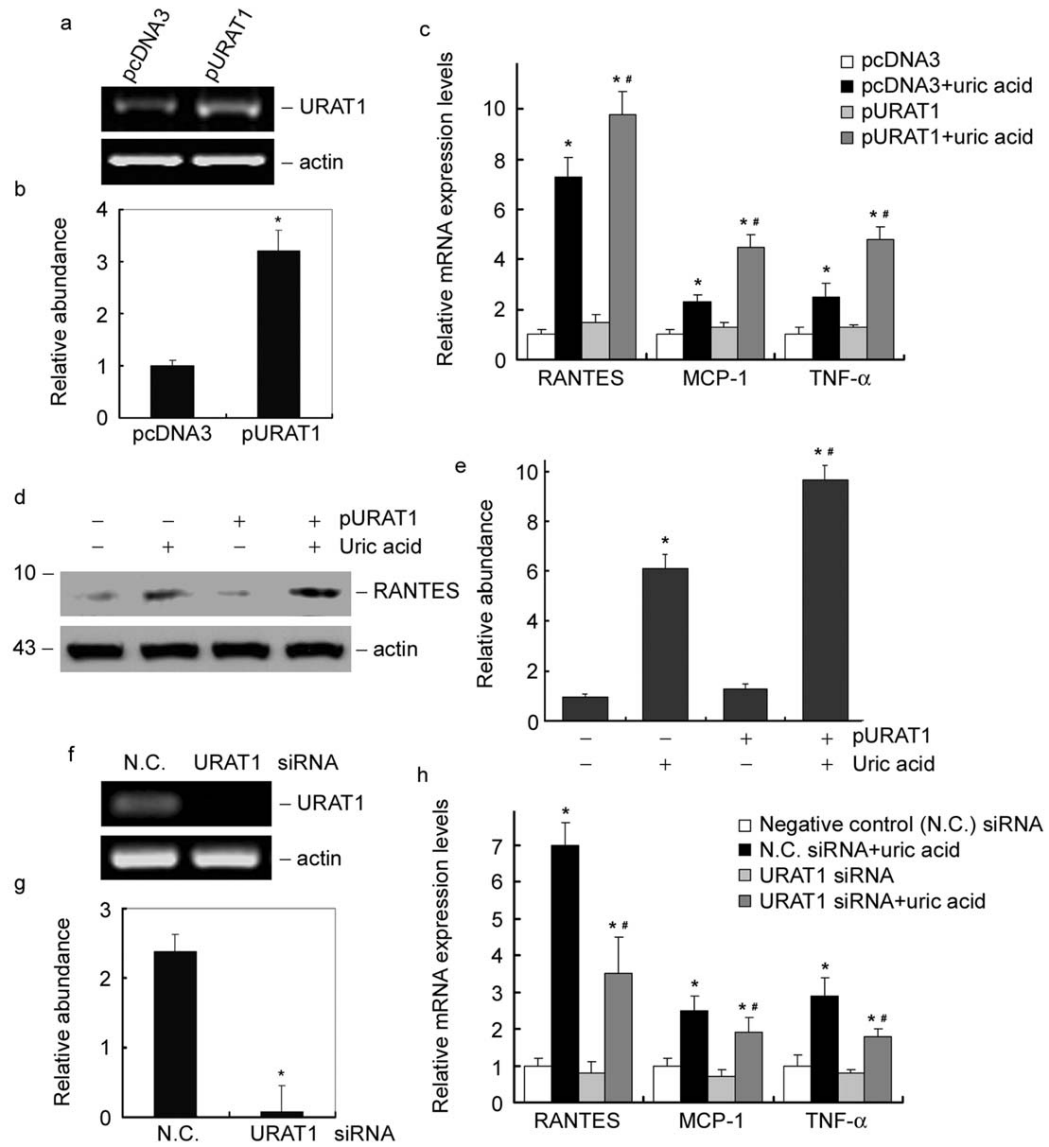
Modulation of urate transporter regulates tubular express of RANTES, MCP-1 and TNF-α in response to uric acid treatment. (a) RT-PCR analysis showed the URAT1 expression level after plasmid transfection for 24h. (b) Graphic presentation of relative mRNA abundance normalized to actin. *P<0.05 versus pcDNA3 transfected group (n = 3). (c) Q-PCR results showed that upregulation of URAT1 by plasmid transfection sensitized tubular express of RANTES, MCP-1 and TNF-α mRNA after 6 h of uric acid treatment. *P<0.05 versus control. #*P*<0.05 versus cells transfected with pcDNA3 (*n* = 3). (d) Western blot results showed that upregulation of URAT1 by plasmid transfection sensitized tubular express of RANTES protein after 24h of uric acid treatment. (e) Graphic presentation of relative RANTES protein abundance normalized to actin. *P<0.05 versus control. #*P*<0.05 versus uric acid treated cells transfected with pcDNA3 (*n* = 3). (f) RT-PCR analysis showed the URAT1 expression level after transfection of URAT1 siRNA for 24h. (g) Graphic presentation of relative mRNA abundance normalized to actin. *P<0.05 versus negative control (N.C.) siRNA transfected group (n = 3). (h) Q-PCR results showed that downregulation of URAT1 by RNA interference partially decreased tubular express of RANTES, MCP-1 and TNF-α mRNA after 6h of uric acid treatment in NRK-52E cells. *P<0.05 versus control. #*P*<0.05 versus uric acid treated cells transfected with negative control siRNA (*n* = 3).

To further confirm the role of uric acid on induction of RANTES, MCP-1 and TNF-α, a pair of reverse strategies was applied. URAT1, one of the human urate transporters located on the apical membrane of tubular epithelial cells, was identified to play important roles in homeostasis, which might sensitize uric acid-induced tubular injury. As shown in figure 5a, expression of URAT1 was up-regulated after 24h of plasmid transfection. After that, cells were incubated with 200μM of uric acid for another 6h. As compared with cells transfected with empty vector (pcDNA3), URAT1 plasmid transfection markedly sensitized tubular express of RANTES, MCP-1 and TNF-α mRNA (figure 5c). After 24h of uric acid treatment, RANTES protein expression was also significantly increased in URAT1-transfected cells (figure 5d). On the contrary, expression of URAT1 was down-regulated after 24h of siRNA transfection (figure 5f). As one of the members of urate transporters on tubular cells, down-regulation of URAT1 (figure 5g) partially decreased tubular express of RANTES, MCP-1 and TNF-α mRNA after 6h of uric acid incubation (figure 5h). These results confirmed the effect of accumulated uric acid on induction of RANTES, MCP-1 and TNF-α. In addition, urate transporters mediated uptaking of uric acid into tubular cells were indispensable in the pathologic conditions.

### Uric acid induces the recruitment of macrophage in vitro

**Figure 6:**
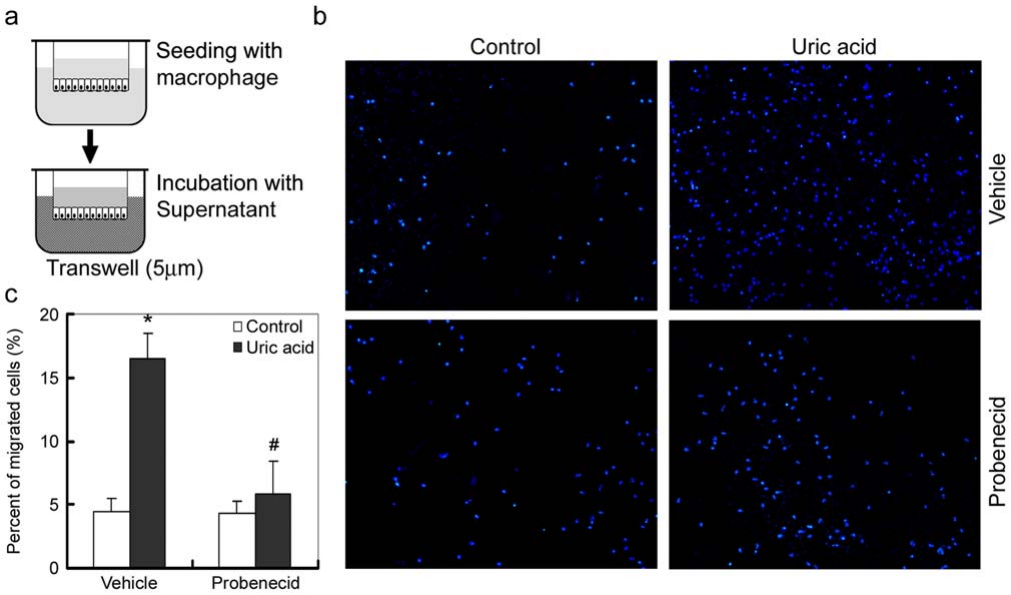
Culture media from uric acid treated tubular cells induce the recruitment of macrophage in vitro. Probenecid reduces the media-induced recruitment of macrophage. (a) Schematic depiction of the chemotaxis assay. Macrophage monolayer on transwell filters was incubated conditioned media from NRK-52E cells treated with or without uric acid for 4 hours, and macrophage migration was then determined. (b) Representative pictures show the migrated macrophage in the bottom chambers of transwell plates in various groups as indicated. (c) For quantification, positive stained nuclei per field (×200) were counted. Ten randomly selected fields were counted. As for the total cell number, ten randomly chose fields (×200) were counted before the chemotaxis assay. Data are expressed as the percentage of migrated cells in total cells added and presented as means ± SEM of three independent experiments. *P<0.05 versus control.

We further examined the chemo-attractive abilities of tubular cells after uric acid incubation by chemotaxis assay. NRK-52E cells were treated with uric acid (200μM) for 24h. The culture media were collected and their abilities to attract macrophage recruitment were assess. Figure 6b and 6c showed that media of uric acid incubated NRK-52E cells were more potent to attract macrophage, compared with control. The chemo-attractive effects of the media were abolished with the presence of probenecid (20μM), which suggested that although uric acid may directly mediate the recruitment of macrophage, uric acid activated cytokines are more potent and critical for attracting macrophage migration.

### Uric acid activates NF-κB signaling pathway in tubular cell

**Figure 7:**
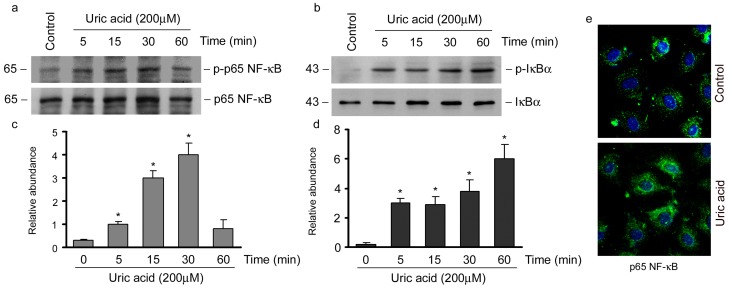
Uric acid activates NF-κB signaling in tubular epithelial cells. (a) Western blot analysis showed that uric acid induced p65 NF-κB phosphorylation and activation in NRK-52E cells. (b) Western blot showed that uric acid induced IκB phosphorylation in NRK-52E cells. (c) Graphic presentation of relative phosphs-p65 NF-κB protein abundance normalized to p65 NF-κB. *P<0.05 versus control (*n* = 3). (d) Graphic presentation of relative phosphs-IκB protein abundance normalized to IκB. *P<0.05 versus control (*n* = 3). (e) Immunofluorescence staining demonstrated that p65 NF-κB underwent nuclear translocation upon uric acid stimulation in NRK-52E cells.

Since NF-κB signaling is important in inflammation, we speculated that RANTES expression may be regulated by NF-κB signaling. We examined the expression of activated p65 NF-κB protein in NRK-52E cells incubated with uric acid for various time periods. As shown in figure 7a, phosphorylation of p65 NF-κB was observed as early as 5min after uric acid incubation. However, the total p65 NF-κB abundance was not altered at different time points. Similar results were obtained for phosphor-IκB-α (figure 7b). The nuclear translocation of p65 NF-κB after uric acid treatment was then examined. Immunofluorescent staining demonstrated that upon uric acid stimulation, p65 NF-κB rapidly translocated into the nuclei of NRK-52E cells (figure 7e).

### NF-κB signaling is critical for RANTES induction in tubular cell

**Figure 8:**
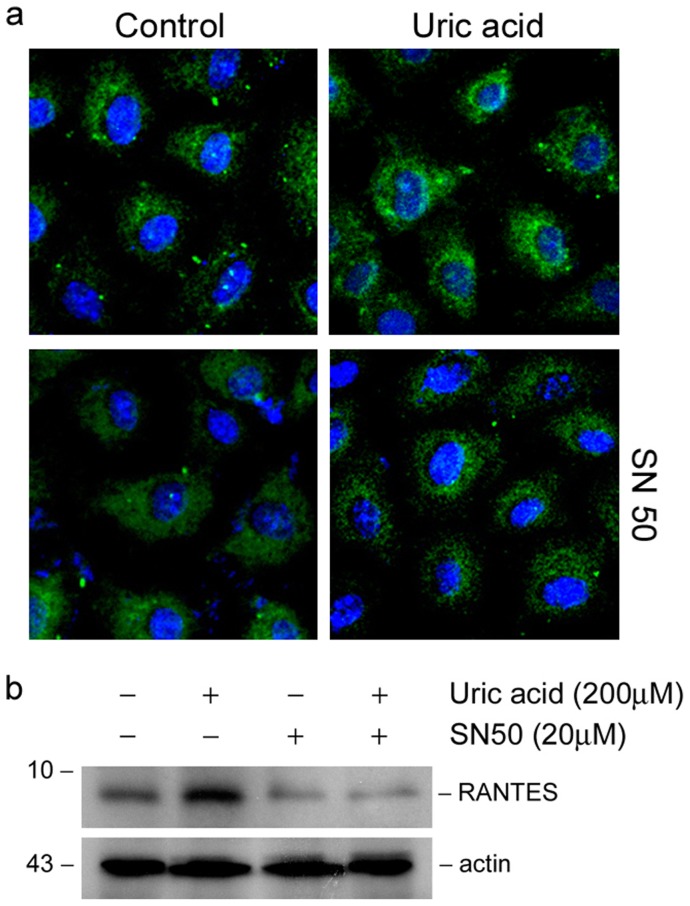
NF-κB signaling is critical for mediating RANTES expression in tubular cells. NRK-52E cells were treated with 200μmol/L of uric acid in the absence or presence of NF-κB inhibitor (NF-κB SN50). (A) Immunofluorescence staining demonstrated that incubation with NF-κB SN50 inhibited nuclear translocation of p65 NF-κB upon uric acid stimulation in NRK-52E cells. (b) Inhibition of NF-κB signaling abrogated RANTES expression stimulated by uric acid in NRK-52E cells.

To test the importance of NF-κB signaling in RANTES induction in tubular cells, we treated NRK-52E cells with uric acid in the absence or presence of specific NF-κB inhibitor (NF-κB SN50), a cell permeable inhibitor peptide which inhibit p65 translocation (figure 8a). As shown in figure 8b, inhibition of NF-κB signaling abolished RANTES expression induced by uric acid, suggesting that NF-κB signaling is required for RANTES induction.

### Uric acid activates NF-κB signaling in vivo

**Figure 9:**
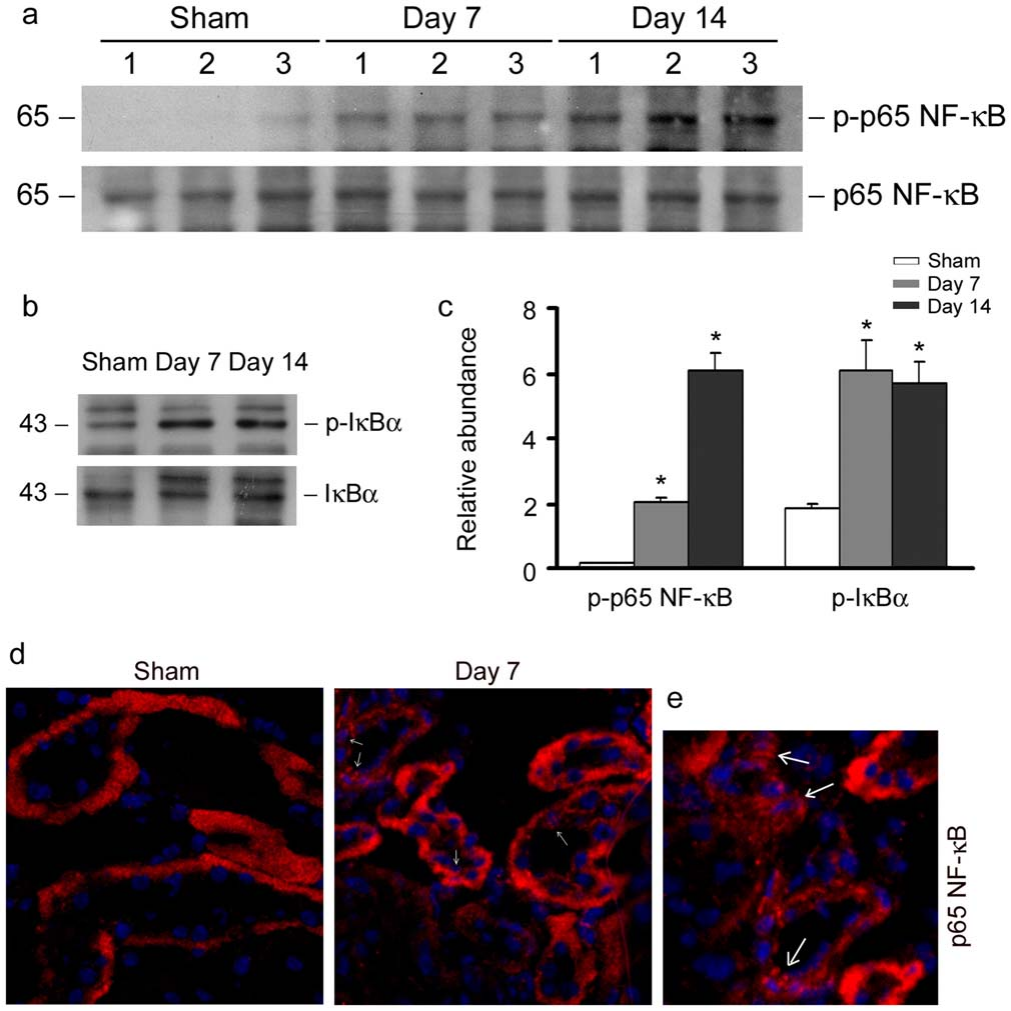
Uric acid activates NF-κB signaling pathway in kidney of hyperuricemia mice. (a) Western blot demonstrated that uric acid induced p65 NF-κB phosphorylation and activation in kidney of hyperuricemia mice. (b) Western blot showed that uric acid induced IκB phosphorylation *in vivo*. (c) Graphic presentation of relative phospho-p65 NF-κB and phospho-IκB protein abundance normalized to p65 NF-κB and IκB, respectively. *P<0.05 versus control (*n* = 3). (d) Immunofluorescent staining demonstrated that p65 NF-κB underwent nuclear translocation in kidney of hyperuricemia mice. Kidney sections were immunostained for total p65 (red) and the nuclei (blue). Arrowheads indicate nuclear staining of p65 NF-κB. (e) Enlarged image shows nuclear translocation of p65 NF-κB in tubules of hyperuricemia mice (day 7) demonstrated by immunofluorescent staining.

We next investigated whether NF-κB signaling was activated in hyperuricemia mice kidney. As shown in figure 9, continuous injection of uric acid markedly activated p65 NF-κB as revealed by western blot analysis of whole-kidney lysates. Immunofluorescence staining demonstrated that p65 NF-κB was localized in the cytoplasm of tubular cells in sham-control kidney; however, uric acid induced p65 NF-κB nuclear translocation apparently, which is similar to uric acid treatment in vitro.

## Discussion

In this study, hyperuricemia mice model were induced by intraperitoneally injection of uric acid (figure 1d) as previously described [15], [17], [18]. Results demonstrate that uric acid induces renal inflammation by recruiting T cells and macrophage infiltration and pro-inflammatory cytokines and chemokines expression in hyperuricemia mice kidneys. NF-κB signaling, a principal signaling pathway mediating pro-inflammatory response in various conditions, was activated in tubule of hyperuricemia mice. Because uric acid is a well-known pro-inflammatory factor and inflammation is a critical mechanism contributing to the progression of renal injury, our study shed new light on understanding the pathogenesis of hyperuricemia renal injury.

Previous investigations showed that fructose, a metabolic source of uric acid, could induce pro-inflammatory mediators in human proximal tubular cells [19]. TNF-α is known to be an important pro-inflammatory cytokine produced by infiltrated cells. It stimulates tubular cells to produce chemokines which, in turn, recruit inflammatory cells. TNF-α is also implicated to be induced and secreted by tubular cells [20]. Increased RANTES expression has been characterized in a variety of kidney disorders, including acute kidney injury and chronic renal fibrosis [21], [22], [23]. In this study, the expression of TNF-α was increased as early as 1h and reached the peak at 24h (figure 3c) in tubular cells, which might create a vicious circuit leading to a sustained chemokines production and inflammatory infiltration in the circumstance of hyperuricemia. On the other hand, RANTES expression was markedly increased and kept at a high level till 24h (figure 3a). The diverse expression patterns might suggest different regulation mechanisms of TNF-α and RANTES in hyperuricemia. The extended effects of TNF-α induction on tubular cells and possible influence on RANTES and MCP-1 expression need further investigations.

In the human kidneys, 90% of filtered urate is reabsorbed via transporters located in the apical membrane of the proximal tubules. Previous investigations showed an up-regulation of URAT1 expression induced by uric acid treatment in proximal tubular cells [15]. The hyperuricosuria caused by hyperuricemia and up-regulation of urate transporter, will probably increase the quantity of uric acid in proximal tubular cells, which aggravates uric acid-induced tubule dysfunction. In this study, up-regulation of URAT1 by plasmid transfection sensitizes tubular express of RANTES, MCP-1 and TNF-α in response to uric acid treatment (figure 5). While blockage of urate transporter by non-specific inhibitor, probenecid, markedly inhibits tubular induction of RANTES, MCP-1 and TNF-α (figure 4). However, blockade of URAT1 only partially abolished RANTES, MCP-1 and TNF-α expression, which might be reasonable as URAT1 is only one of numerous transporter proteins in epithelial cells. Blockage of URAT1 could not abolish the uptake of uric acid mediated by other transporters. These results further confirmed the effect of accumulated uric acid on induction of RANTES, MCP-1 and TNF-α, which also suggest that urate transporter mediated uric acid accumulation in tubule plays important role in mediating tubule inflammation.

Chemokines and cytokines play fundamental roles in recruitment and activation of effecter inflammatory cells. As inflammatory cells are no longer the only cells contribute to development of inflammation, tubular epithelial cells, the largest cell population in kidney parenchyma, are important players in renal inflammation after injury. Tubular production of RANTES and MCP-1 inevitably contributes to the formation of a chemo-attractive microenvironment, which attract the infiltration of T cells and macrophages into tubular interstitial spaces [20], [24]. In this study, as shown by chemotaxis assay, although uric acid may directly has chemo-attractive effect on macrophages, tubular expression and secretion of RANTES and MCP-1 stimulated by uric acid are more potent and critical for attracting macrophage migration (figure 6).

In this study, uric acid induces renal inflammation via NF-κB signaling. Transcription factor NF-κB is silenced by its specific inhibitor, IκB, in normal resting state. Upon activation, after phosphorylation and degradation of IκB, NF-κB is released and translocated in to nucleus to dictate the transcription of its target genes. RANTES is one of the genes controlled by NF-κB. The data presented in this study suggest that uric acid activates NF-κB signaling in both hyperuricemia mice kidneys and tubular epithelial cells. Moreover, RANTES expression stimulated by uric acid is NF-κB dependent (figure 8). Although the protective effect of NF-κB inhibition *in vitro* is clear, it remains to be determined whether this therapeutic strategy is operative *in vivo* for ameliorating renal inflammation. In addition, the possibility that uric acid regulates inflammatory mediator expression by signaling mechanisms other than NF-κB could not be excluded, as blockage of p65 NF-κB translocation only partially abolish TNF-α and MCP-1 induction (data not shown), which suggested that TNF-α and MCP-1 were not totally regulated by NF-κB. Meanwhile, recent studies suggested that uric acid crystals engaged NALP3 inflammasome to induce tubular-interstitial inflammation [10], [25]. Furthermore, NF-κB is an important transcription factor that regulates genes involved in immune development [26], synaptic plasticity and memory [27]. Glucocorticosteroid has already been administered for gout patients; however, there remains a long distance towards the administration of NF-κB inhibitor in clinical settings.

In summary, we showed in this study that uric acid induced infiltration of inflammatory cells and production of inflammatory mediators in hyperuricemia mice. These pro-inflammatory effects may be mediated by activation of NF-κB signaling. Therefore, in addition to cause arthritis in gout, uric acid also causes inflammation via NF-κB signaling in kidney, which might provide a therapeutic target in clinical settings.

## Methods

### Ethics statement

All of the following details of the study were approval by institutional review board of Nanjing Medical University.

### Animals

Male CD-1 mice weighing 18–22 g were acquired from Shanghai Experimental Animal Center of Chinese Academy of Science. They were housed in the animal facilities of Center for Kidney Disease of Nanjing Medical University with free access to food and water and treated in compliance with the regulations and protocols of institutional review board of Nanjing Medical University. Mouse hyperuricemia model was generated by daily intraperitoneally injection of uric acid (Sigma) (250mg/kg). [15], [17], [18] For the sham-control group, normal saline was administered. Groups of mice (*n* = 5) were killed at 7 and 14 d, respectively, and the kidneys were removed for various analyses.

### Cell culture and treatment

Normal Rat Kidney epithelial cells (NRK-52E) were obtained from cell resource center of Shanghai Institutes for Biological Sciences Chinese Academy of Sciences, which was originally came from ATCC (CRL-1571TM). Cell culture and uric acid treatments were carried out according to the procedures described previously [15]. Briefly, NRK-52E cells (1.0×10^6^) were seeded in DMEM-F12 medium that contained 10% FBS at approximately 80% confluence. After an overnight incubation, cells were serum-starved in serum-free medium for 24 h before addition of sterile uric acid. The concentration of uric acid applied in this study was 200μM. For blocking the kidney urate transporter, NRK-52E cells were incubated with probenecid (P8761, Sigma-Aldrich) at a concentration of 20 μM. For blocking NF-κB signaling, NRK-52E cells were pretreated with NF-κB SN50 (Calbiochem), a cell-permeable inhibitor peptide, at a concentration of 20 μM for 1 h and then incubated with uric acid. Whole-cell lysates or conditioned media were prepared and then subjected to various analyses.

### Plasmids transfection

URAT1 expression plasmid was kindly provided by 301 Hospital of PLA, Beijing, China. The empty expression plasmid vector pcDNA3 was purchased from Invitrogen (San Diego, CA, USA). Plasmids DNA (4μg in 2ml medium) were transiently transfected into cells using Liptofectamine 2000 transfection reagent (Invitrogen) according to the protocols provided by the manufacturer. Twenty-four hours after transfection, cells were used for experiments.

### URAT1 knockdown by siRNA transfection

SiRNA and negative control siRNA (acquired from Ambion) (100 pmol/2ml medium without antibiotics) were transfected into cells using Lipofectamine 2000 reagent (Invitrogen) according to the protocols provided by the manufacturer and then incubated at 37°C in a CO2 incubator for 24h until the cells were ready for assay.

### Western blot analysis

The preparation of whole-cell lysates and kidney tissue homogenates and western blot analysis of protein expression were carried out using previous routine procedures [28]. The primary antibodies used were as follows: Anti-RANTES (sc-1410; Santa Cruz Biotechnology); anti-phospho p65 NF-κB (Ser536) (93H1) (3033; Cell Signaling Technology), anti-p65 NF-κB (3034; Cell Signaling Technology), anti-phospho IκB-α (Ser32) (14D4) (2859; Cell Signaling Technology), anti-IκB-α (44D4) (4812; Cell Signaling Technology) and anti-actin (A5385; Sigma Aldrich). Quantification was performed by measurement of the intensity of the signals with aid of National Institutes of Health Image software package.

### Quantitative polymerase chain reaction

Quantitative polymerase chain reaction (Q-PCR) was performed using an Applied Biosystems 7300 Sequence Detection system. The CT data were determined using default threshold settings and the mean CT was determined from the duplicate PCRs. The ratio to sham-control group was calculated by using the equation 2^-ΔCT^, in which ΔCT  =  CT _Sample_-CT _control_. All the primers were acquired from QIAGEN.

### Reverse transcriptase-polymerase chain reaction analysis

Total RNA was prepared using a TRIzol RNA isolation system according to the instructions by the manufacturer (Invitrogen). The first strand of cDNA was synthesized using 2 μg of RNA in 20 μl of reaction buffer using MLV-RT (Promega, Madison, WI) and random primers at 42°C for 30 minutes. PCR was performed using a standard PCR kit on 1μl aliquots of cDNA and HotStarTaq polymerase (Promega, Madison, WI) with specific primer pairs. The sequences of primer pairs were as follows: URAT1(forward) 5′- GTC TTC ACT GGG CAG CTT CTG-3′ and (reverse) 5′- CAA ACA GGT ATG GCC AGG TAC TC-3′; β-Actin (forward) 5′-CAG CTG AGA GGG AAA TCG TG-3′ and (reverse) 5′-CGT TGC CAA TAG TGA TGA CC-3′. The PCR products were size fractionated on a 1% agarose gel and detected by NA-green (D0133, Beyotime) staining. Quantification was performed by measurement of the intensity of the signals with aid of National Institutes of Health Image software package.

### Immunohistochemical and Immunofluorescence Staining

Immunohistochemical staining of kidney sections was performed according to an established protocol [28]. In brief, paraffin-embedded sections were stained with anti-CD3 (sc-20047; Santa Cruz Biotechnology) and anti-CD68 (ab995; Abcam) antibodies using the Vector M.O.M. immunodetection kit, according to the protocol by the manufacturer (Vector Laboratories, Burlingame, CA). Indirect immunofluorescence staining was carried out according to the procedures described previously [28]. Briefly, cells or kidney cryosections were incubated with the specific primary anti-p65 NF-κB antibody (3034; Cell Signaling Technology), followed by staining with cyanine Cy3–conjugated secondary antibody (Jackson ImmunoResearch Laboratories). Cells were double stained with DAPI to visualize the nuclei. Slides were viewed with a Nikon Eclipse E80i microscope equipped with a digital camera (DS-Ri1, Nikon). CD3 and CD68 positive cell numbers per high-power field (×400) were counted. Ten randomly selected field of each kidney were counted.

### Morphological Studies

Tissue sections from the mice were prepared at 3μm thickness by a routine procedure. Sections were stained with hematoxylin/eosin for general histology. Slides were viewed with a Nikon Eclipse E80i microscope equipped with a digital camera (DS-Ri1, Nikon).

### Chemotaxis assay

Cell chemotaxis assay was performed using 24-well transwell plant following the procedure as described previously [20]. Macrophages (5×10^5^ in 100μl) were freshly prepared from mouse peritoneal fluid and added onto the upper chamber of the transwell insert (5μm; Corning). NRK-52E conditioned media (0.5ml) were added in the lower chamber. After incubation at 37°C for 4h, cells were stained with DAPI and viewed with microscope and counted. Data are expressed as percentage of the migrated cells in total number of input cells. For quantification, positive stained nuclei per field (×200) were counted. Ten randomly selected fields were counted. As for the total cell number, ten randomly chose fields (×200) were counted before the chemotaxis assay.

### Statistical analysis

Statistical analysis was performed using SigmaStat software (Jandel Scientific Software). Comparisons between groups were made using one-way ANOVA, followed by the *t* test. *P*<0.05 was considered significant.
